# Supplementary figures and images for: Tolerance to Haemophilus influenzae infection in human epithelial cells: Insights from a primary cell-based model
Source: PLoS Pathog. 2024 Jul 11;20(7):e1012282. doi: 10.1371/journal.ppat.1012282 (PMC11239077; doi:10.1371/journal.ppat.1012282)

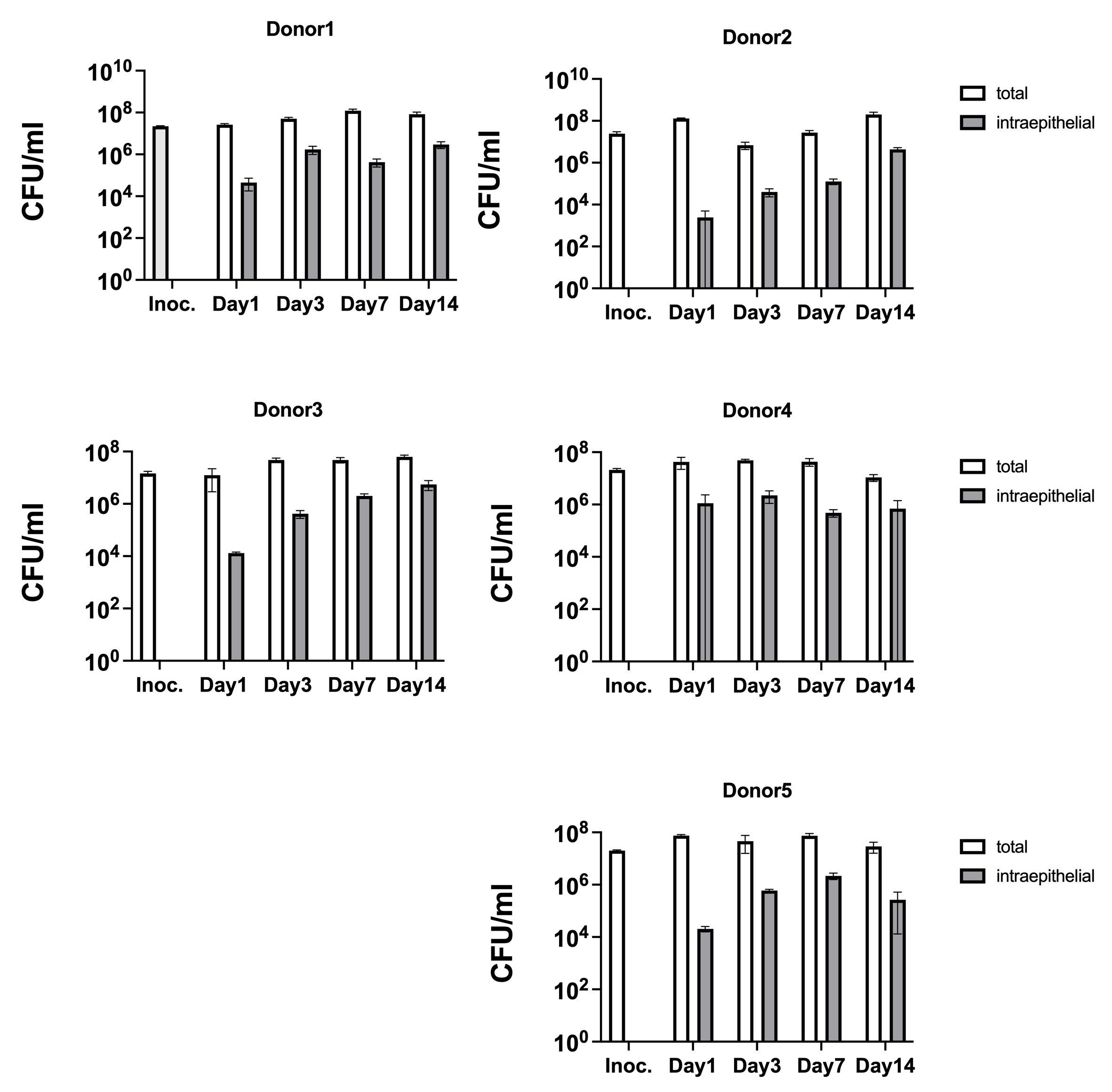

Supplement: S1 Fig — Bacterial Loads in in NHNE from different donors. Inoc.–inoculum. (TIFF) [file ppat.1012282.s001.tiff]

**Panel A:**

Day1

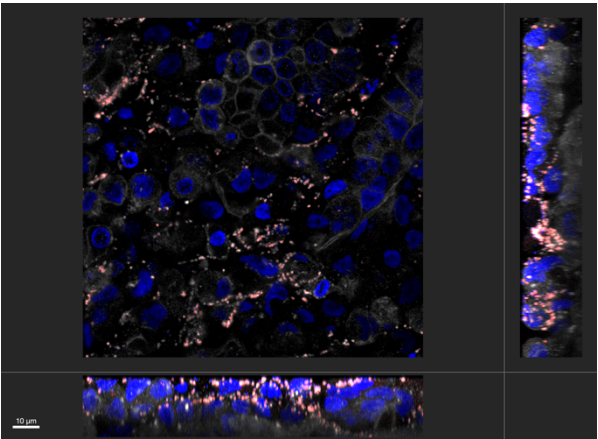

Day 3

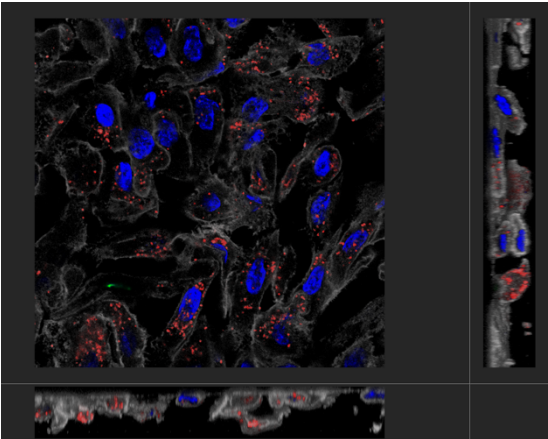

Day 7

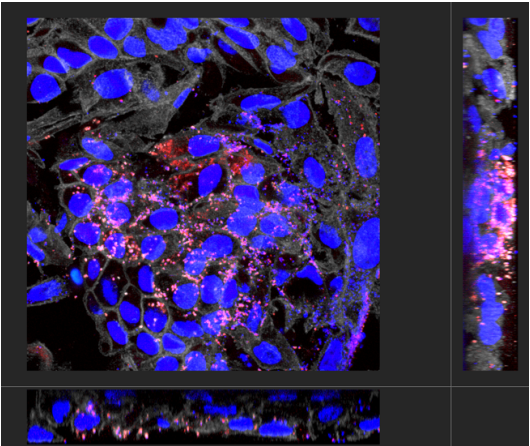

Day14

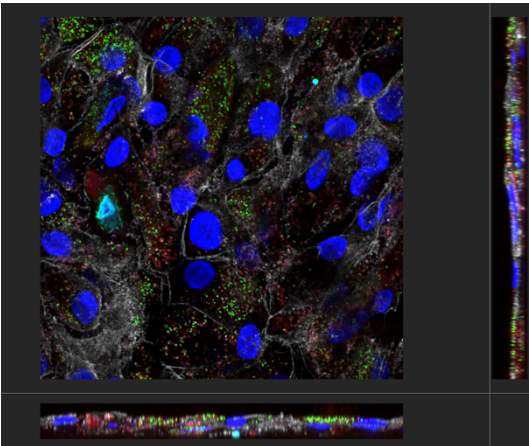

Uninfected

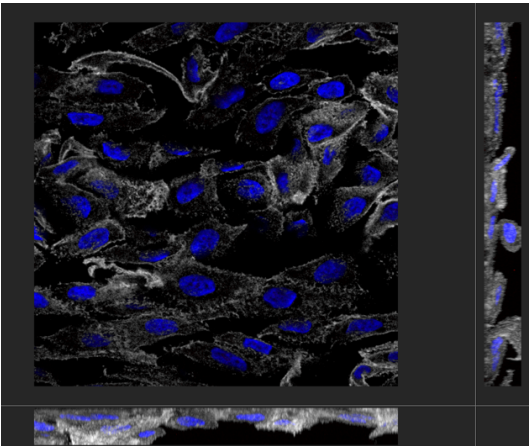

**Panel B:**

Day1

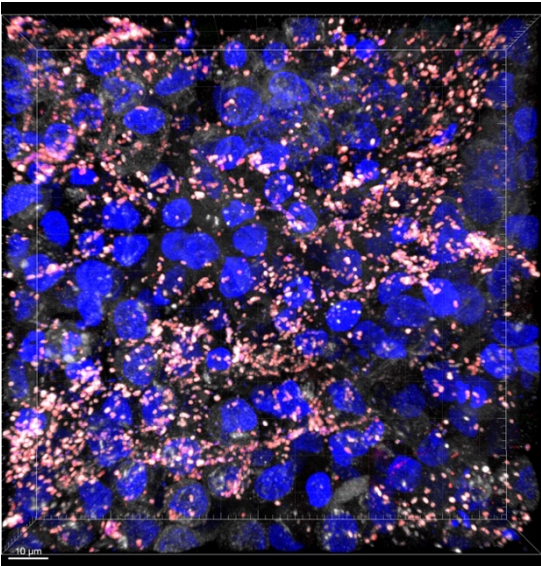

Day3

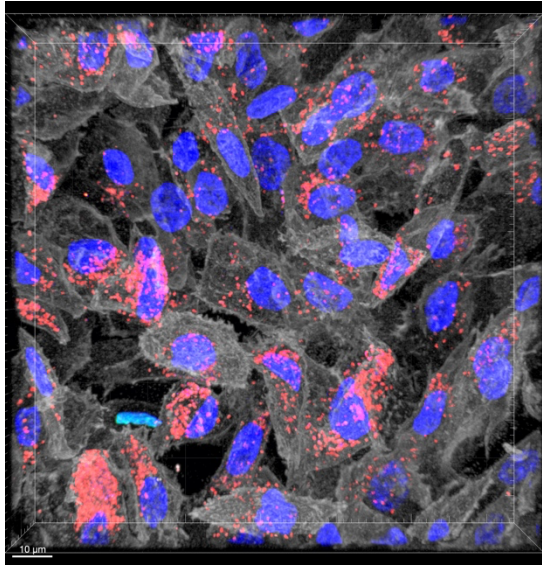

Day7

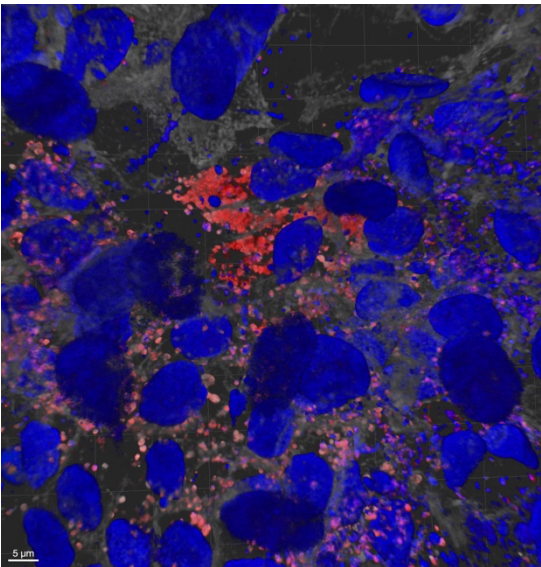

Day14

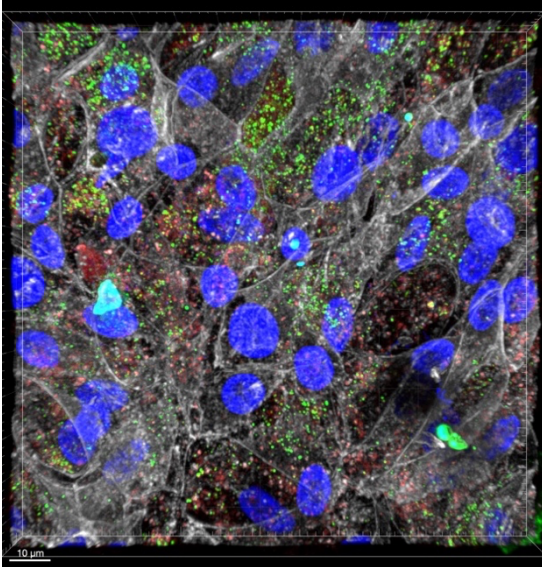

uninfected

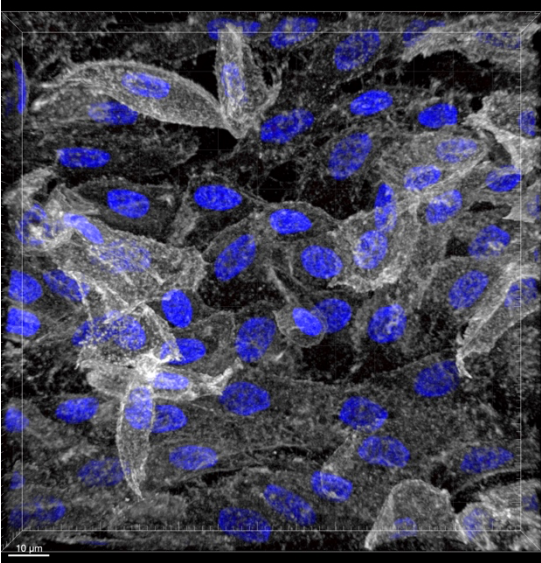

Supplement: S2 Fig — Panel A: Confocal microscopy images with orthogonal projections. Panel B: Confocal microscopy image 3D projections, top view. Images were generated using Imaris Viewer Software (Oxford Instruments). Staining used NTHi anibodies (red), Phalloidin–cellular junctions and tubulin (white), Nuclei–DAPI stain (blue), DNA-breaks/ apoptosis–TUNEL probe (green). (PDF) [file ppat.1012282.s002.pdf]

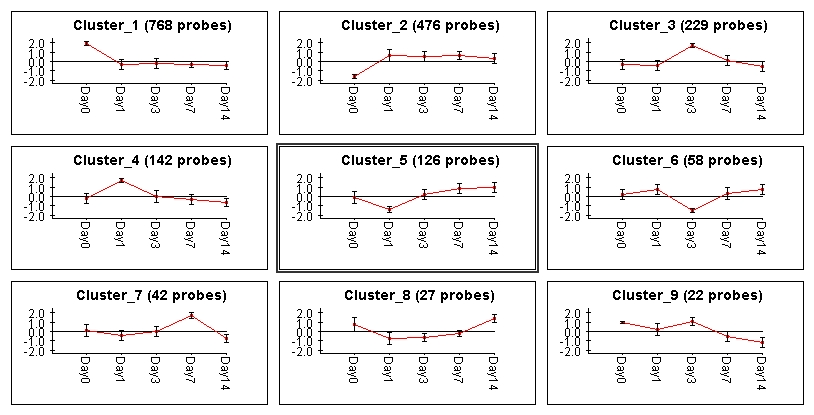

Supplement: S3 Fig — Gene expression clusters (fold-changes) identified for NTHi using the CLICK algorithm integrated into the EXPANDER package. Using default settings, nine clusters were identified, with only 44 genes not mapped. Clusters 1,5,7 & 9 show a decrease in gene expression from day 0 to Day1. Genes in Cluster 3 also showed this feature but to a lesser degree. Cluster homogeneity values were between 0.803 and 0.883. (TIFF) [file ppat.1012282.s003.tiff]

A

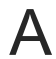

## Da

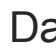

## Da

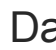

B Day1-Day0

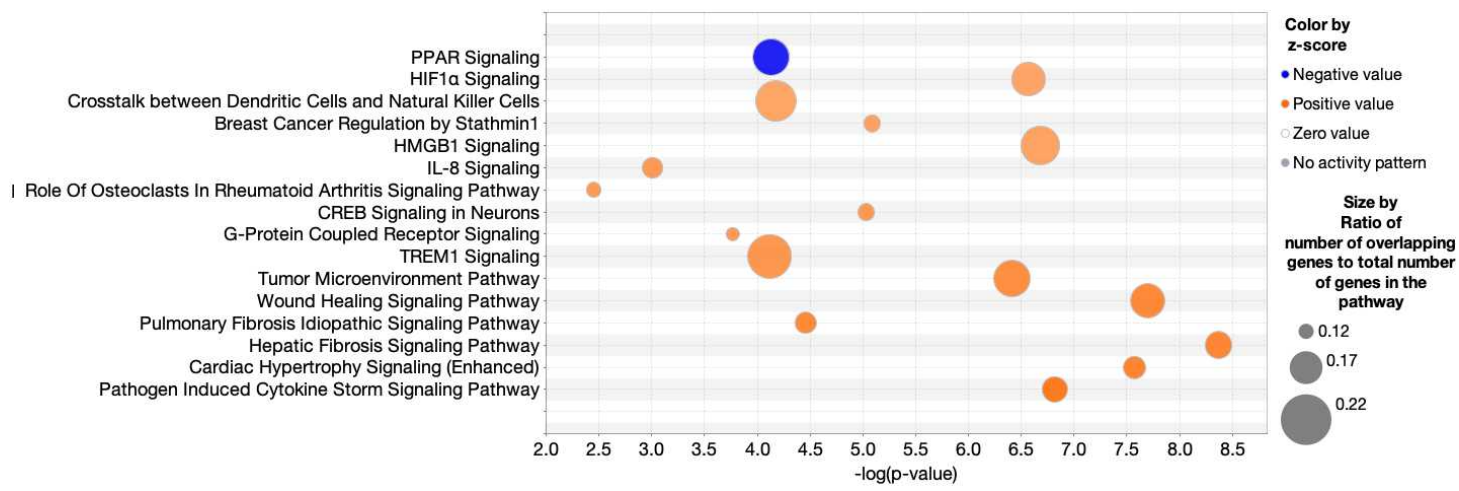

Day3-Day1

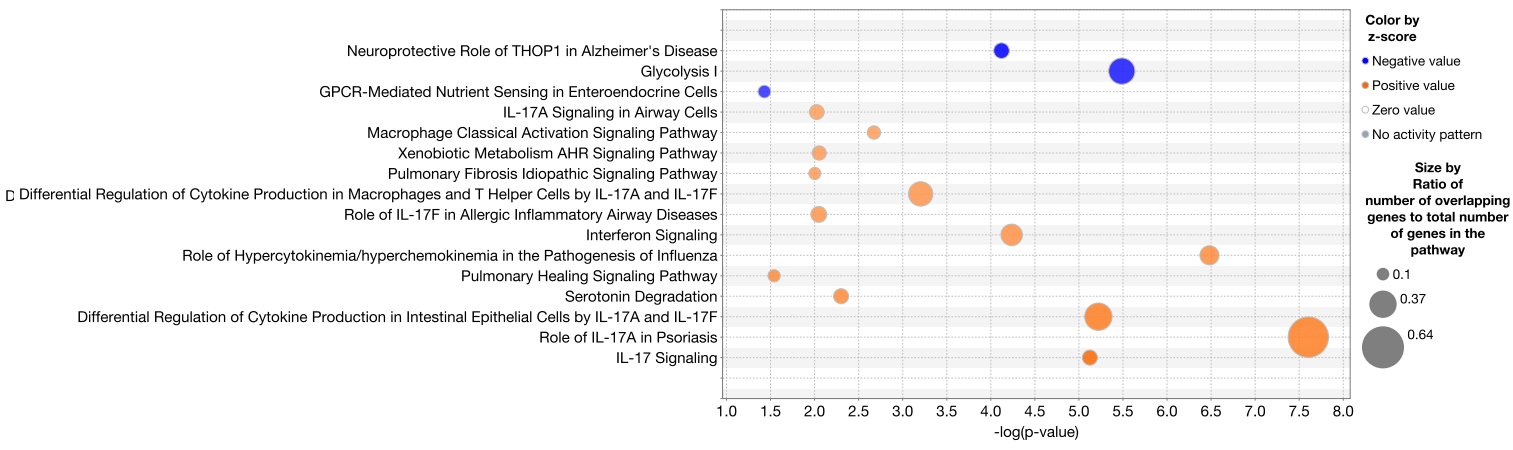

Day7-Day3

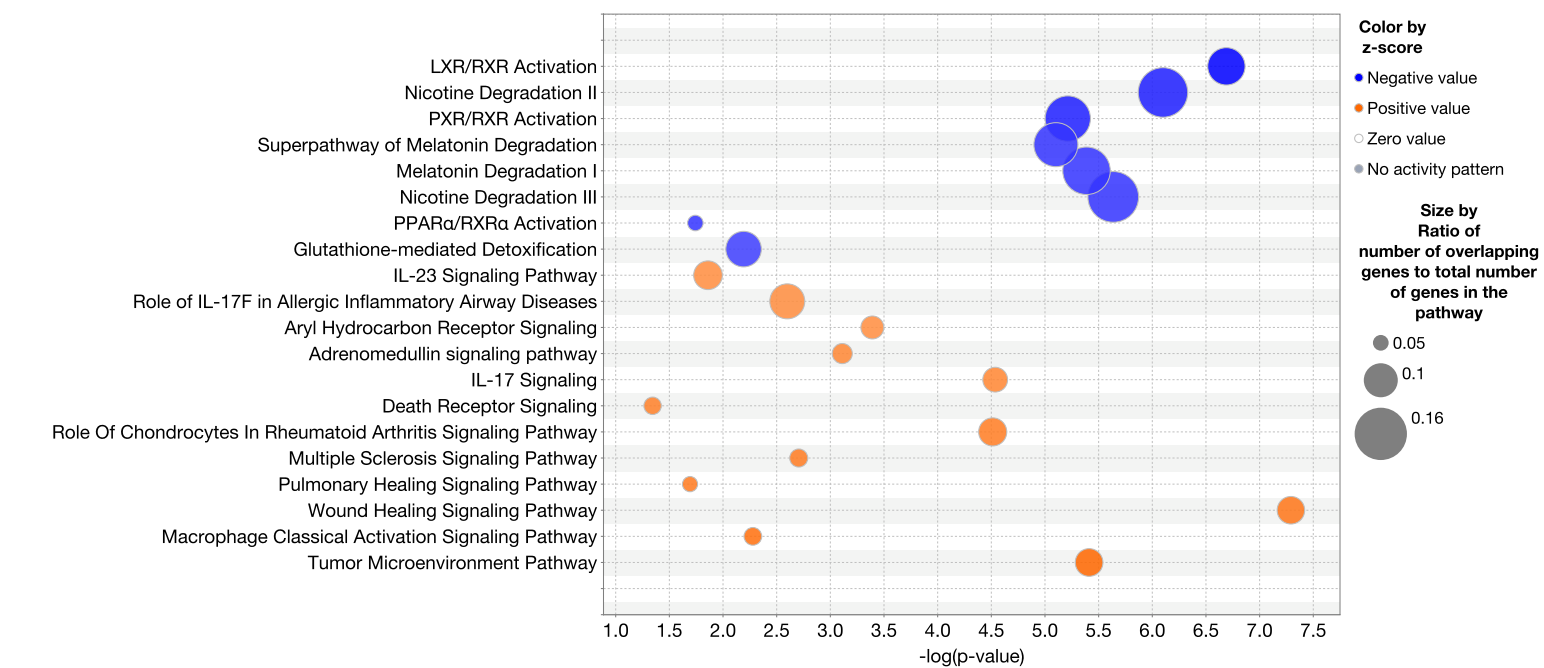

C

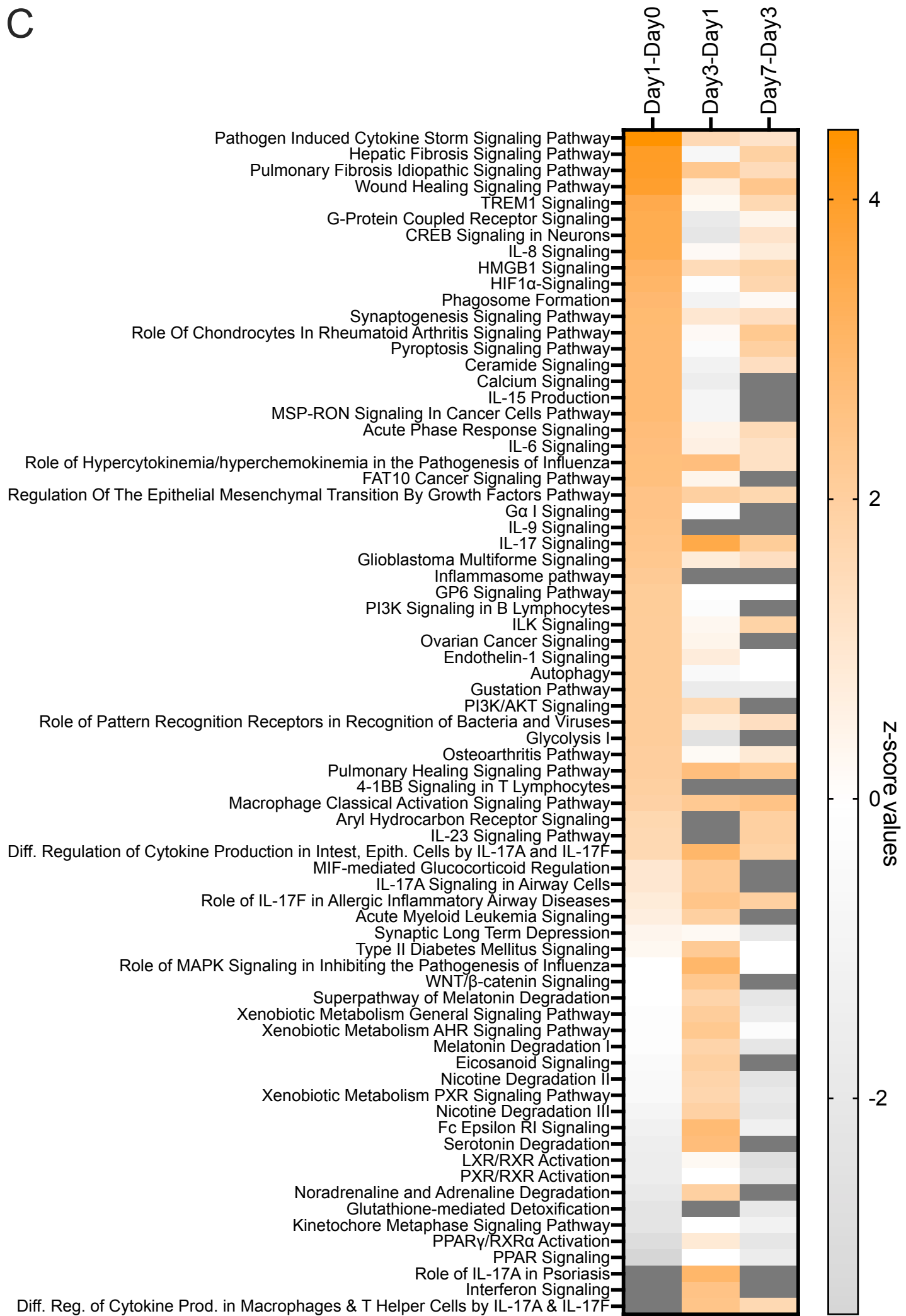

D

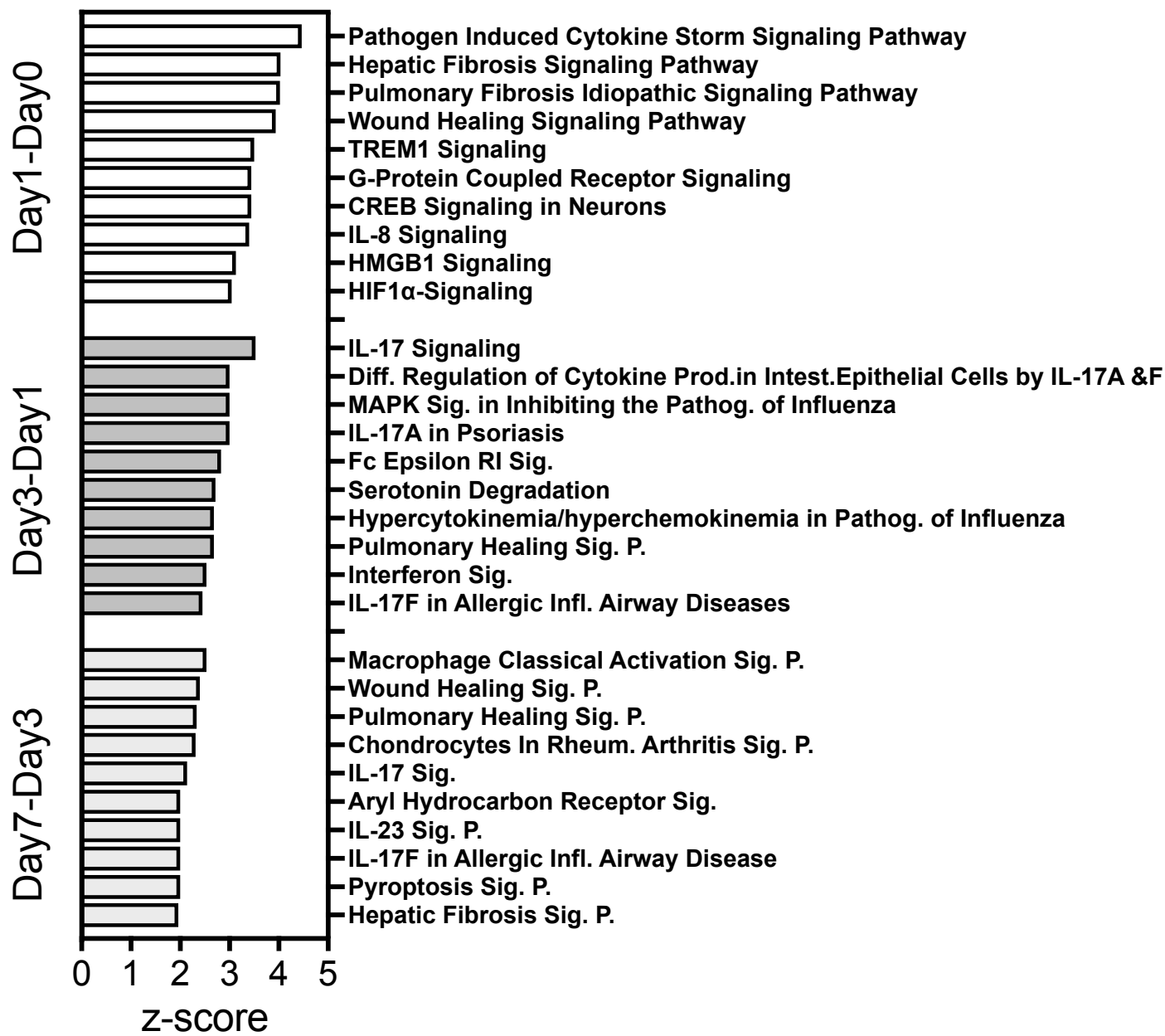

Supplement: S4 Fig — Panel A: Effector summary networks. Panel B: Top Canonical Pathways identified in data comparisons displayed as bubble plots. Z-score cut offs: Day1-Day0: 3.0; Day3-Day1: 2.2, Day7-Day3: 2.0, FDR p-value <0.05. Panel C: Heatmap of canonical pathways with z-score of at least 2 in one of the three comparisons. Data is sorted in descending order for the Day1-Day0 data. Dark gray colour—pathway not identified. Panel D: Top 10 Canonical Pathways and their z-scores. (PDF) [file ppat.1012282.s004.pdf]

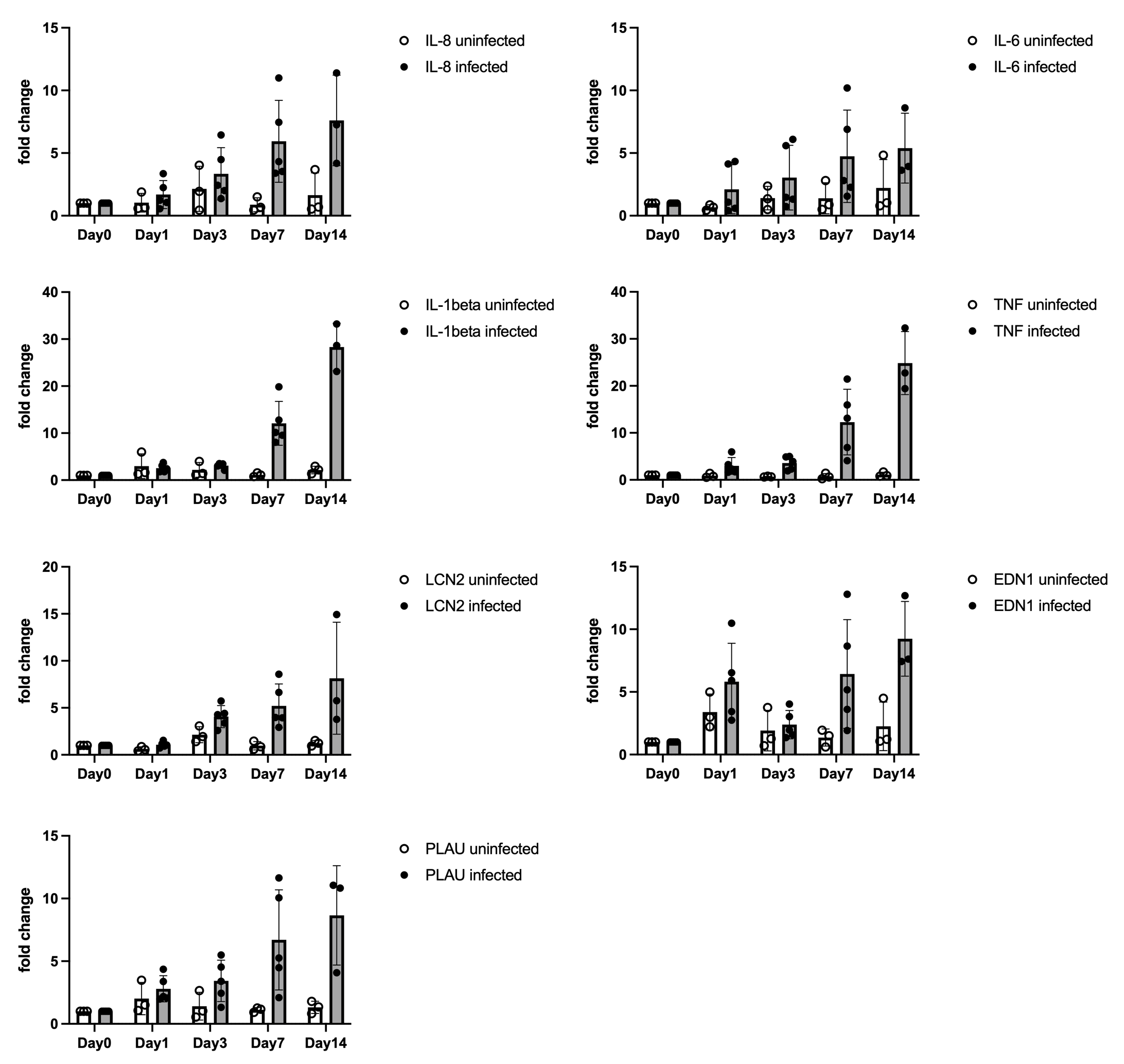

Supplement: S5 Fig — Data from qPCR (uninfected samples) and RNAseq (infected samples were normalized against the day zero value. Each datapoint is derived from values for between 3 and 5 biological replicates. Data are shown as averages, error bars represent standard deviation. (TIFF) [file ppat.1012282.s005.tiff]

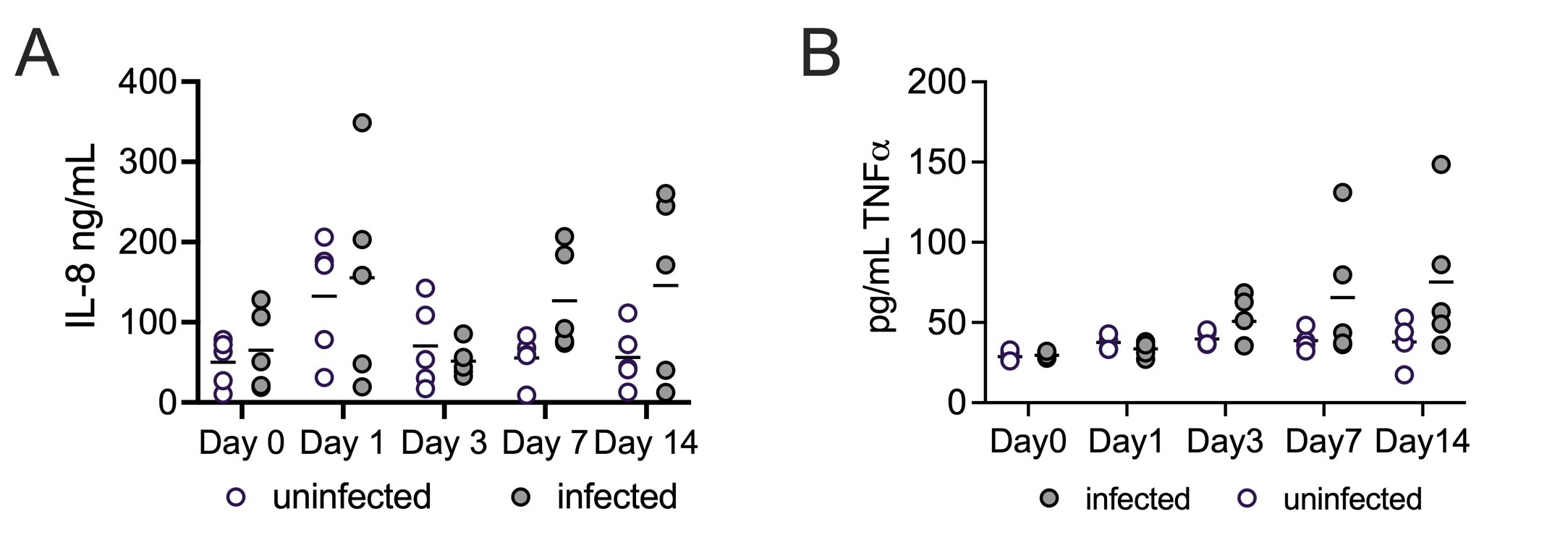

Supplement: S6 Fig — Detection of IL-8 (A) and TNFα (B) in NHNE apical wash fluid using ELISA. Each datapoint (shown as a circle) represents an average of at least n = 3 replicate wells for each donor. (TIFF) [file ppat.1012282.s006.tiff]

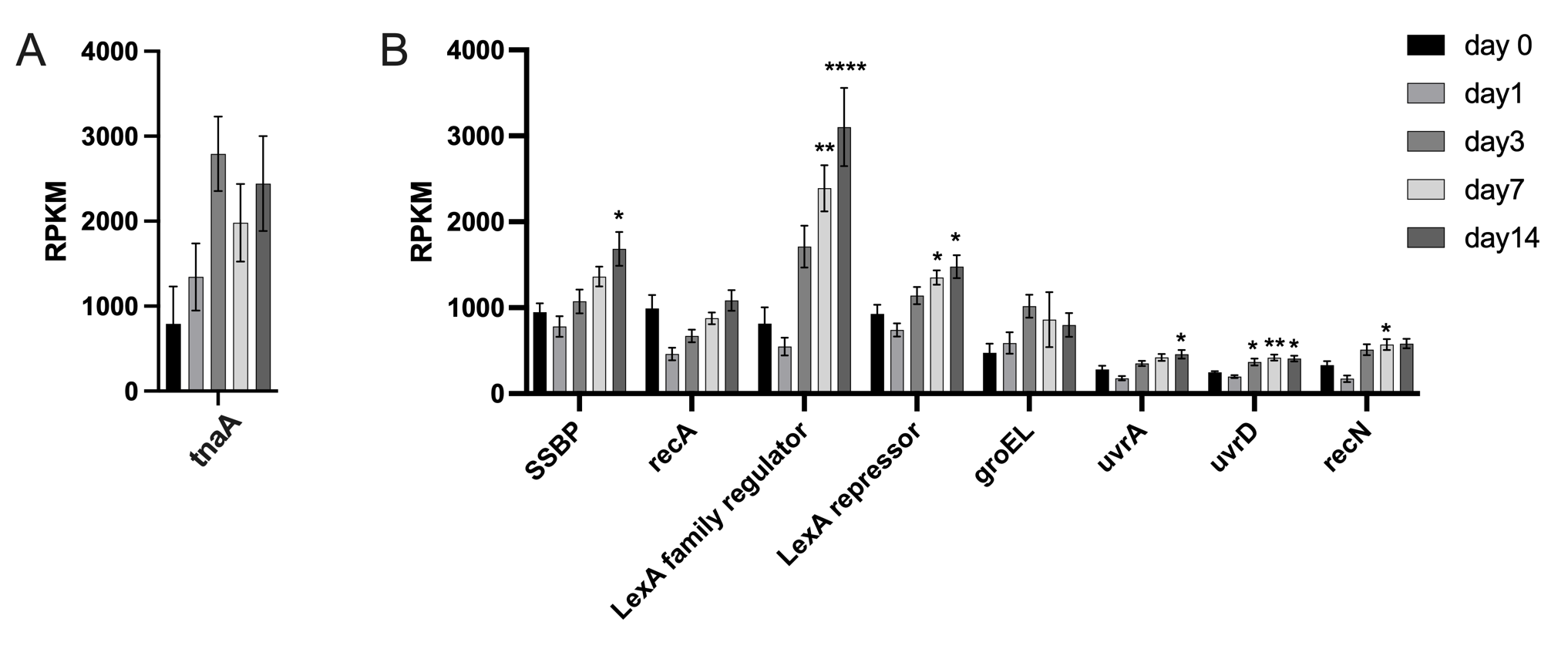

Supplement: S7 Fig — Panel A: tnaA tryptophanase, Panel B: genes involved in SOS responses. RPKM values shown are averages of values obtained from n = 5 donors (Donors 1–5), except for the Day14 values (n = 3, Donors 1–3). Statistical analyses used 1-Way ANOVA, using the Day0 value as the reference. p-values: *<0.05, **<0.01, **** <0.0001. (TIFF) [file ppat.1012282.s007.tiff]

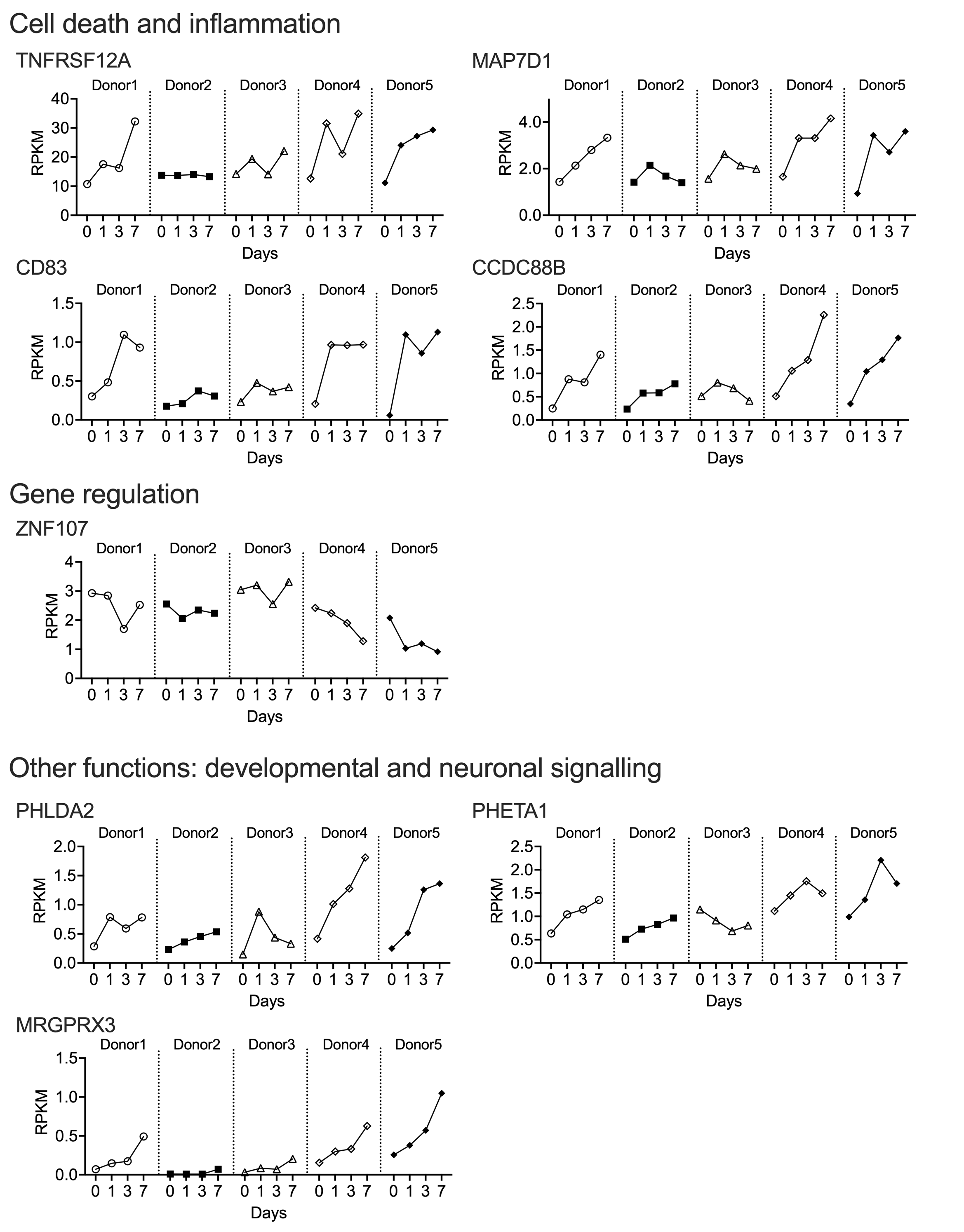

Supplement: S8 Fig — RPKM patterns are shown for each donor from Day0—Day7. Only genes with a max RPKM or > 1 are shown. All genes had Pearson coefficients with an absolute value of at least 0.75. (TIFF) [file ppat.1012282.s008.tiff]

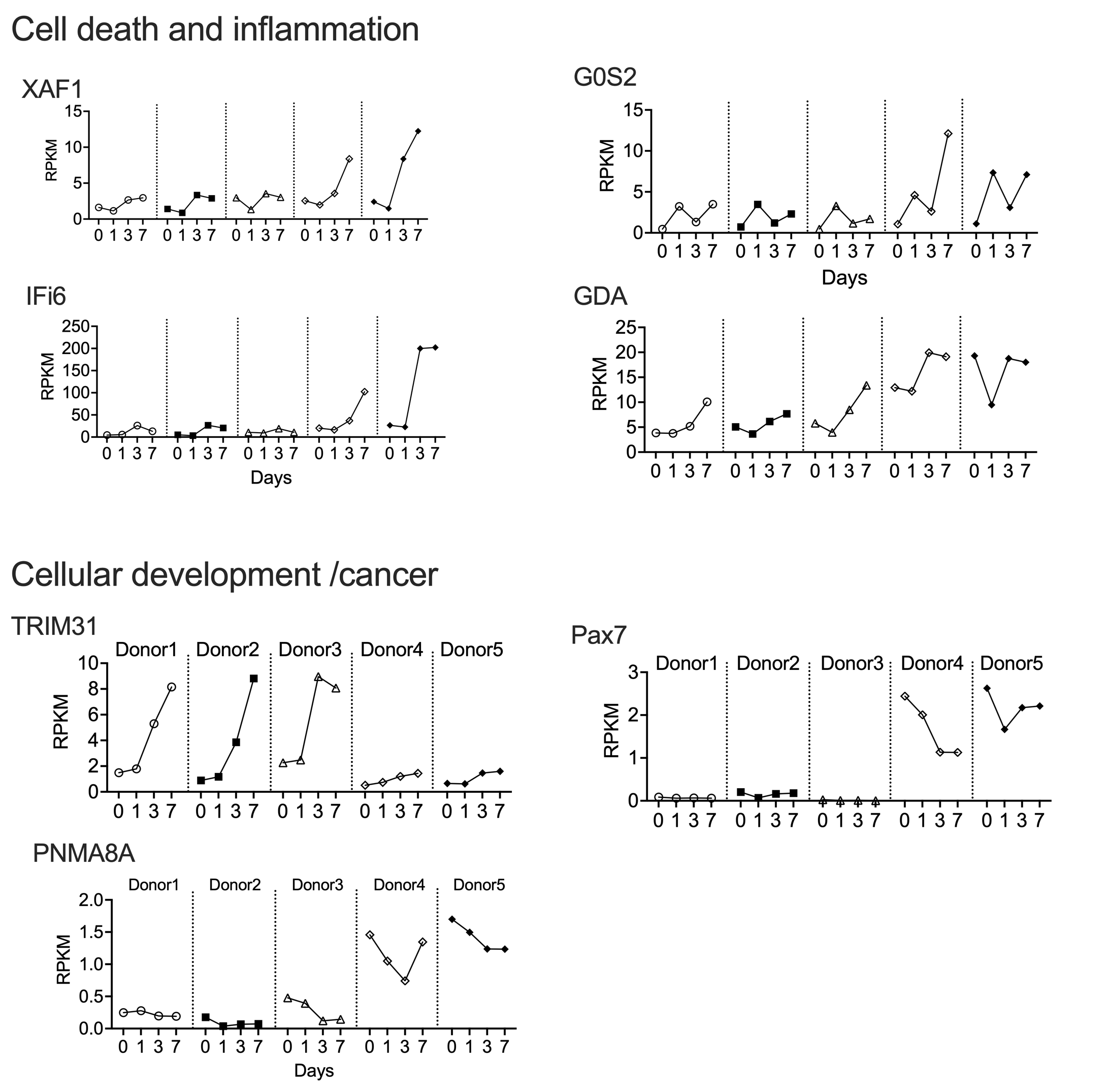

Supplement: S9 Fig — RPKM patterns are shown for each donor from Day0—Day7 (Day14 for donors 1–3). DEGs were identified using the DEG1 tool within the idep.94 platform. (TIFF) [file ppat.1012282.s009.tiff]

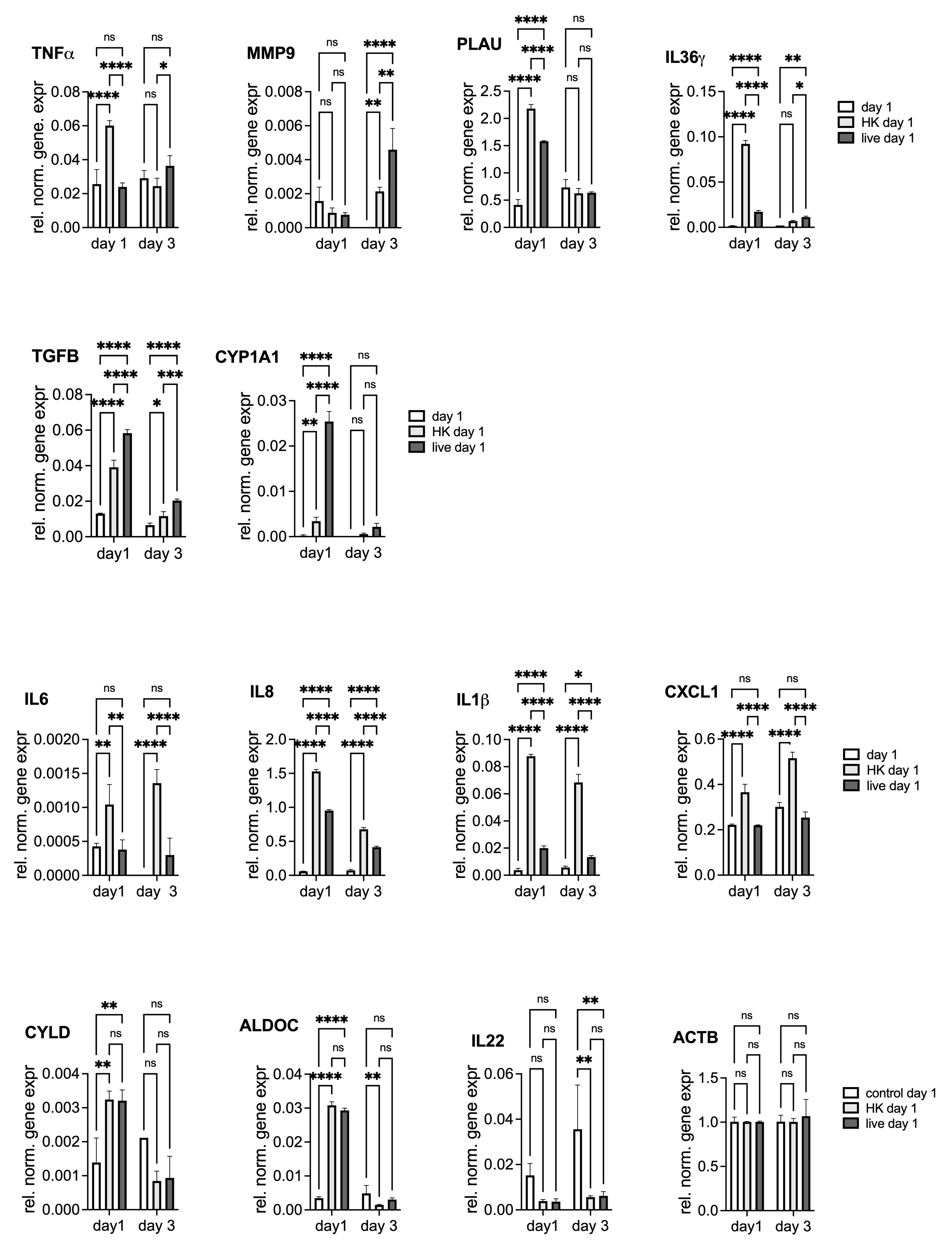

Supplement: S10 Fig — Gene expression values were normalized to expression of ACTB. Statistical testing used 2-Way ANOVA with Tukey’s Multiple Comparison test. * p<0.05, ** p<0.01, *** p<0.001, **** p<0.0001. (TIFF) [file ppat.1012282.s010.tiff]

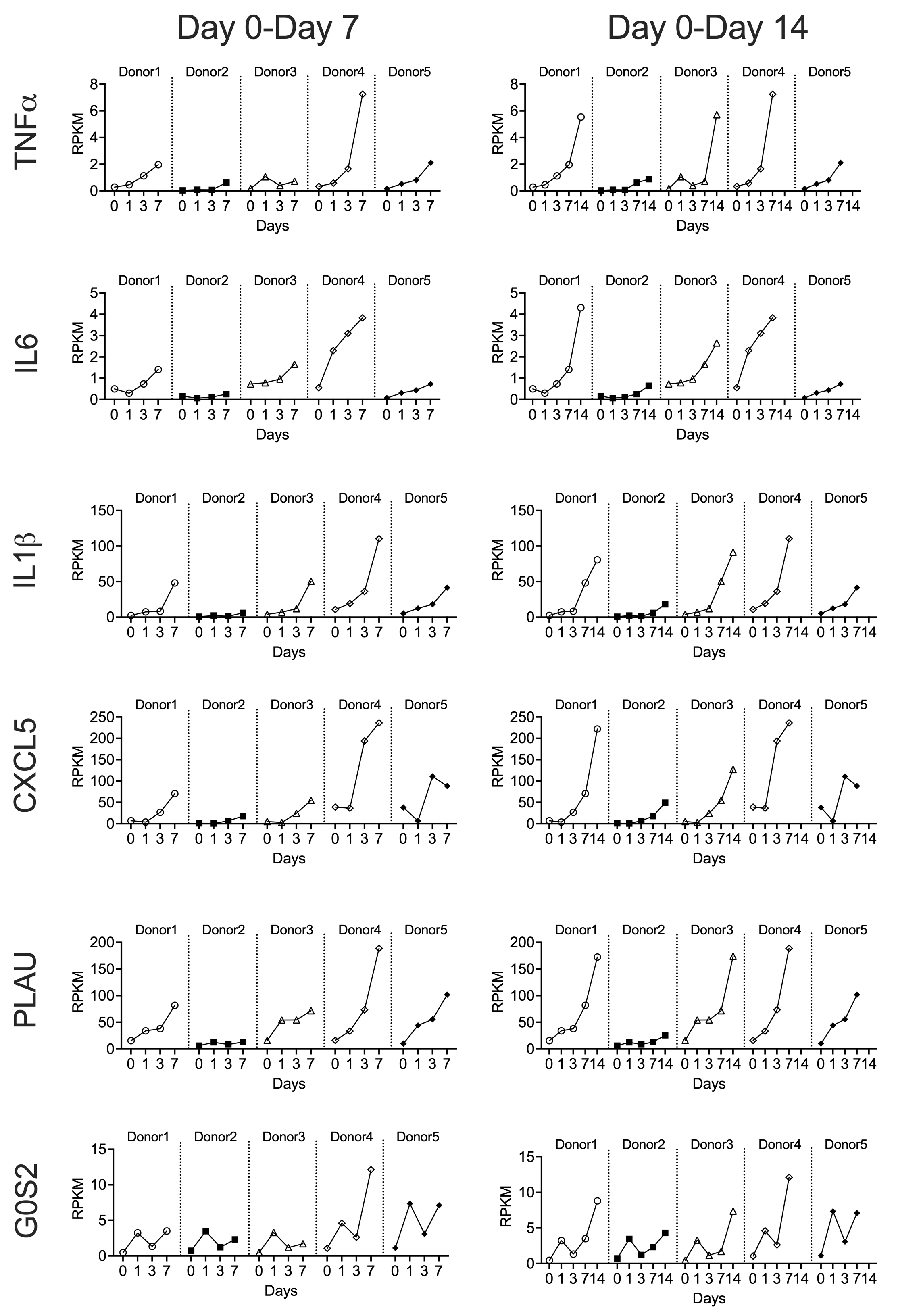

Supplement: S11 Fig — Left diagrams show expression values from Day0 to Day7, right diagrams include RPKM values for Day14 for donors 1–3, highlighting that when considering this extra datapoint, gene expression patterns across donors look more similar than in the Day0- Day7 comparison. (TIFF) [file ppat.1012282.s011.tiff]

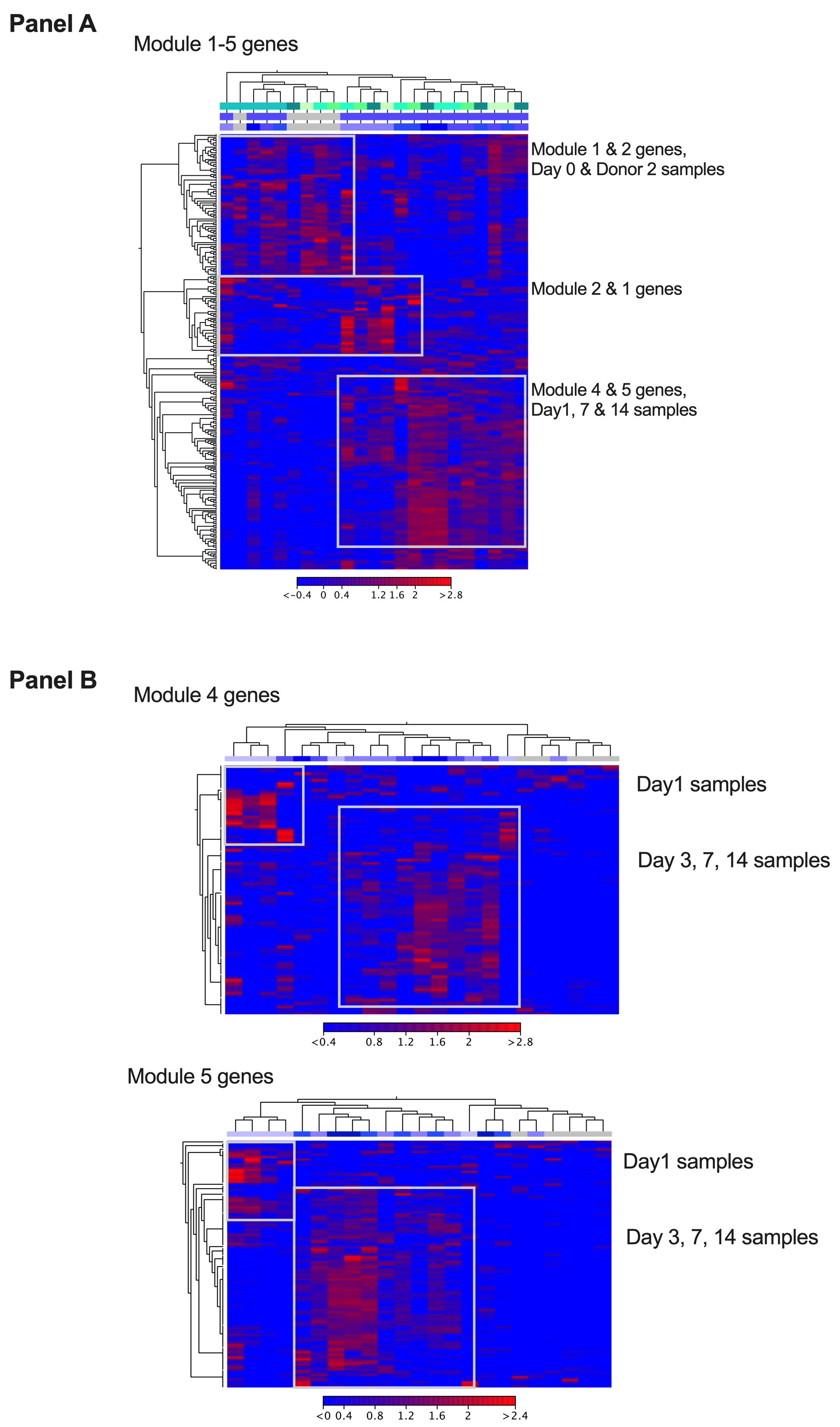

Supplement: S12 Fig — A: Heatmap using genes for all five gene expression modules identified by Avital et al [54] B: Heatmap for module 4 and module 5 genes. Colour bars (top of heatmaps): Heatmap A: top: green colours–donors, middle: grey–uninfected, blue–infected, bottom: sampling time, grey Day 0, blue colours: lightest shade–day 1, Darkest shade—day 14. Heatmap B: sampling time, grey Day 0, blue colours: lightest shade–day 1, Darkest shade—day 14. (TIFF) [file ppat.1012282.s012.tiff]

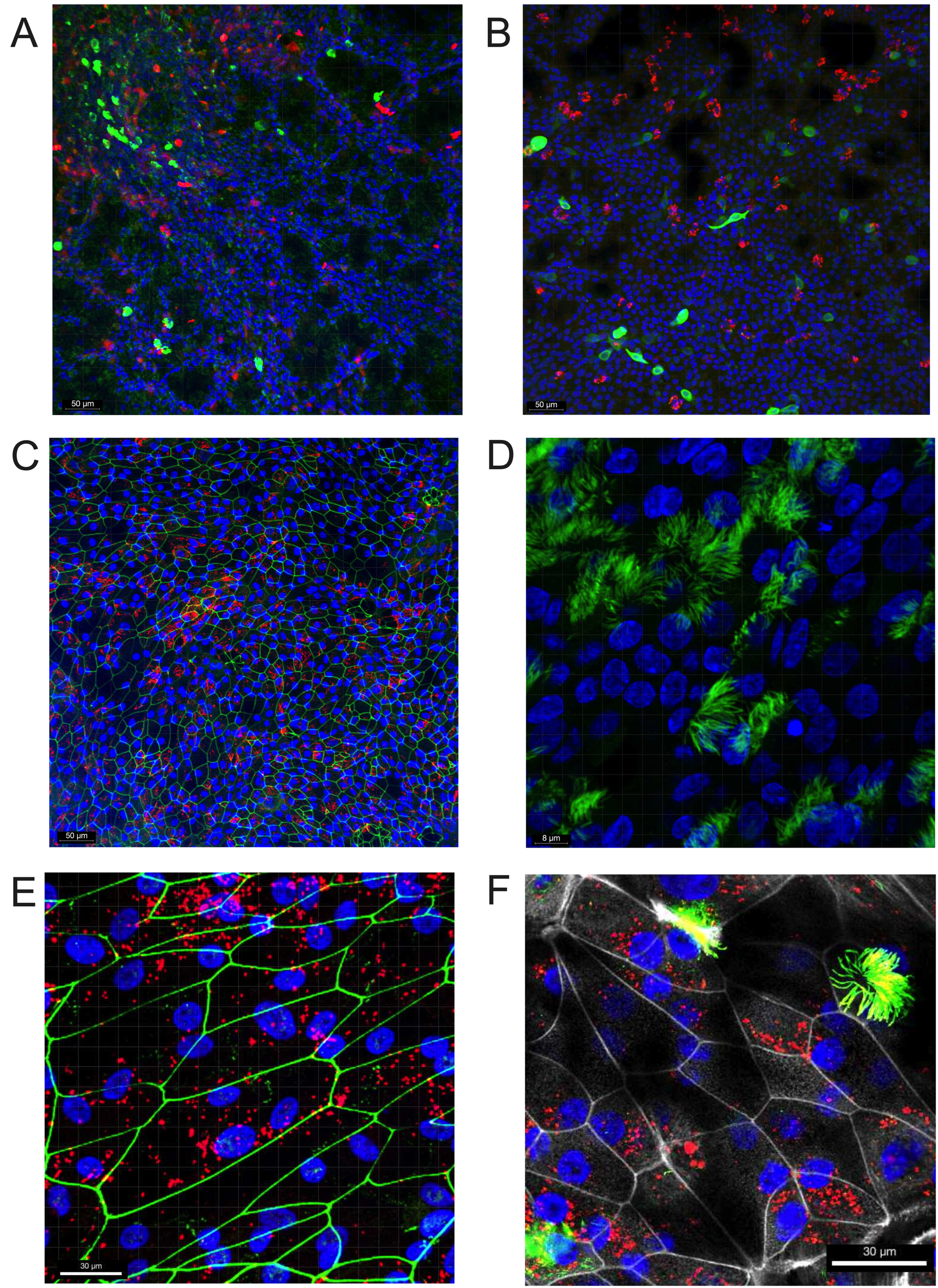

Supplement: S13 Fig — Panel A: NHNE stained for cilia and basal cells. Blue–nuclei (Hoechst), red–basal cells, anti-cytokeratin 14 guinea pig (ab192694, Abcam), green—cilia, monoclonal beta-tubulin IV (T7941, Sigma). Panel B: NHNE stained for cilia and goblet cells. Blue–nuclei (Hoechst), red–cilia, monoclonal beta-tubulin IV (T7941, Sigma), green—anti-MUC5B rabbit (HPA008246, Sigma). Panel C: NHNE stained for cilia and cellular junctions. Blue- nuclei (Hoechst), green–junctions, anti-ZO-1 rabbit 402200 (Thermo Fisher), red—cilia, monoclonal beta-tubulin IV (T7941, Sigma). Panel D: NHNE stained for cilia. Blue–nuclei (Hoechst), green–cilia, monoclonal beta-tubulin IV (T7941, Sigma),. Panel E: NTHi-infected NHNE with ZO-1 staining. Blue—nuclei (Hoechst), green–junctions, anti-ZO-1 rabbit 402200 (Thermo Fisher), red—Anti-NTHi 86-028NP OMP antibodies, gift from Prof. L. Bakaletz, Ref. 58. Panel F: NTHi-infected NHNE with phalloidin staining. Blue- nuclei (Hoechst), green–cilia, monoclonal beta-tubulin IV (T7941, Sigma), white–Phalloidin—647 (Thermo Fisher), red—Anti-NTHi 86-028NP OMP antibodies, gift from Prof. L. Bakaletz, Ref. 58. Panels A-C: 20x magnification, Panel D: 120x magnification, Panels E&F: 60x magnification. (TIFF) [file ppat.1012282.s013.tiff]
