# Supplementary material for: Tolerance to Haemophilus influenzae infection in human epithelial cells: Insights from a primary cell-based model
Source: PLoS Pathog. 2024 Jul 11;20(7):e1012282. doi: 10.1371/journal.ppat.1012282 (PMC11239077; doi:10.1371/journal.ppat.1012282)
Supplement: S6 Table — (DOCX) [file ppat.1012282.s019.docx]

| Primer name | sequences |
| --- | --- |
| HS-QP-ACTB-R | CCAGACGCAGGATGGCATGG |
| HS-QP-ACTB-F | TGCTATCCCTGTACGCCTCTGGC |
| HS_QP_IL8_F | GGTGCAGTTTTGCC AGGAGTGCTA |
| HS_QP_IL8_F | GCGCAGTGTGGTCCACT TCAATC A |
| HS_QP_IL6_F | TGCCCCAGTACCCCCAG AGAAGAT |
| HS_QP_IL6_R | TAGGGCTGAGATCCGTCAGGATG |
| HS_QP_IL1Beta_F | CCAGTACGAATCTCGACCACCA |
| HS_QP_IL1Beta_R | TTCTCCTGGAAGGTCTGTGGGCAG |
| HS_QP_PLAU_F | CTCTTCAGCTGGGCCTGGG |
| HS_QP_PLAU_R | GCGCAGTCATGCACCATGC |
| HS_QP_TNFAIP3_F | CAAGCTGCACGGACTCCTGG |
| HS_QP_TNFAIP3_R | TCTGTACTCGATGAAACACAGTG |
| HS_QP_cyp1a1_F | GAGGTCCTGATAAGCACGTTGC |
| HS_QP_cyp1a1_R | ATAGCGCCGGCCAAAGCAAAT |
| HS_QP_aldoc_F | TGGATGGGCTCTCAGAACGCTGT |
| HS_QP_aldoc_R | CGTTCTCCAGAATGGCAAGTG |
| HS_QP_TNFav2_F | CGTGGAGCTGGCCGAGGAGG |
| HS_QP_TNFav2_R | GGCAGAAGAGCGTGGTGGCG |
| HS_QP_EDN1_F | GGTGGGTGAGAACGGCGG |
| HS_QP_EDN1_R | GTCCAGGTGGCAGAAGTAGACA |
| HS_QP_LCN2F | TCAGACCTGATCCCAGCCCC |
| HS_QP_LCN2R | TCTGAGAATTGCATTCCCTGCC |

Other primer:

| HS-QP-TGFβ-F | TGTGGCTACTGGTGCTGAC |
| --- | --- |
| HS-QP-TGFβ-R | TGGACAGGATCTGGCCGC |
| HS_QP_cxcl10_F | CTTCTACTTTGTACAGTCTTTCATT |
| HS_QP_cxcl10_R | GGGCCCAAATTCTTTCAGTGGCT |
| HS_QP_mmp9_F | CCTCCAACCACCACCACACC |
| HS_QP_mmp9_R | GCCAGCTGAGGGGGGACCTG |
| HS_QP_IL36G_F | CTTTACAGTCACTGTTGCTGTTAT |
| HS_QP_IL36G_R | ACCTTCTCACAATACAAACACATTT |
| HS_QP_cyld_F | CAGAGCAGTTGAATGGCTGGG |
| HS_QP_cyld_R | TTATAACAGGTTTAAGATTCCTGC |
| HS_QP_CXCL1_F | CCCAGCAATCCCCGGCTC |
| HS_QP_CXCL1_R | CAGGGTCTGCAAGCACTGGC |
| HS-QP-IL22-F | CTGCTATCTGATGAAGCAGG |
| HS-QP-IL22-R | CTGTTGCTGAGCCTGGCC |
